# Health behavior of young patients with ischemic stroke in Estonia: A score of five factors

**DOI:** 10.1002/brb3.2908

**Published:** 2023-02-14

**Authors:** Minni Saapar, Riina Vibo, Siim Schneider, Liisa Kõrv, Sandra Mallene, Janika Kõrv

**Affiliations:** ^1^ Department of Neurology and Neurosurgery University of Tartu Tartu Estonia; ^2^ Department of Neurology North Estonia Medical Centre Tallinn Estonia

**Keywords:** Estonia, health behavior, ischemic stroke, risk factors

## Abstract

**Background:**

Behavioral risk factors are common among young patients with stroke. This study aimed to compare the health behavior of patients and healthy controls and develop a combined risk score of health behavior.

**Methods:**

The health behavior of patients aged 18–54 years who suffered an ischemic stroke from 2013 to 2020 in Estonia was compared to the Health Behavior among Estonian Adult Population 2014 study sample. We chose five risk factors for comparison: smoking status, body mass index, physical exercise, diet (salt use and vegetable consumption), alcohol intake (quantity and frequency), and composed a summary score.

**Results:**

Comparing 342 patients and 1789 controls, daily smoking (49.0% vs. 22.7%), obesity (33.4% vs. 15.9%), low physical activity (< twice/week) (72.2% vs. 60.5%), excessive salt use (8.6% vs. 4.5%), and frequent alcohol use (≥ weekly) (39.9% vs. 34.0%) were more prevalent among patients. The differences in infrequent vegetable consumption (<6 days/week) and excessive alcohol consumption (7 days, >8 units/females, >16 units/males) were not significant. The observed differences were similar for age groups 18–44 years and 45–54 years. The average Health Behavior Stroke Risk Score (0–10) was 4.6 points (CI 4.4–4.8, SD ± 1.97) for patients and 3.5 points (CI 3.4–3.6, SD ± 1.90) for controls.

**Conclusions:**

Before stroke, young patients displayed significantly worse health behavior than the general population. The largest differences were found for smoking and obesity, and a cumulation of risk factors was observed via the HBSR score.

## Graphical abstract

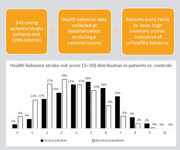

## 1 INTRODUCTION

Lifestyle choices have a significant impact on health, and many studies have emphasized the connection between modifiable risk factors and stroke. It has been demonstrated that more than 80% of strokes are related to hypertension, current smoking status, obesity, unhealthy diet, and physical inactivity (O'Donnell et al., 2010) and that 74% of the stroke burden is due to behavioral factors (Feigin et al., 2016). These traditional stroke risk factors are also common in young patients with stroke (Putaala, 2016), and they are surprisingly prevalent regardless of the stroke etiology (Maaijwee et al., 2014). This may be related to the reported increase in stroke incidence in young adults over the last decades (Ekker et al., 2019). In Estonia, the incidence and case fatality of stroke in young adults are higher than in other high‐income countries (Kõrv et al., 2021), and the traditional risk factors are common (Schneider et al., 2017; Vibo et al., 2021). Previous studies on young patients with stroke mainly focused on clinical risk factors, and only a few have evaluated health behavior. More complex topics, for example, diet, are often not included, and there is no standard evaluation of all the factors.

## 2 OBJECTIVES

The present study aimed to assess the health behavior of young patients with stroke, compare this to the general population, and develop a simple health behavior summary score to evaluate composite behavioral stroke risk.

## 3 METHODS

The Estonian Young Stroke Registry is a prospective hospital‐based ongoing database, which comprises all consecutive patients with a discharge diagnosis of ischemic stroke (94.9% first‐ever, 6.1% recurrent) and aged 18−54 years, as described previously (Vibo et al., 2021). While hospitalized for acute stroke, written informed consent was obtained from the participants, and they completed a self‐report questionnaire about their health behavior before the stroke. The questionnaire was designed to match the Health Behavior among Estonian Adult Population (HBEAP) study of 2014. The HBEAP study is conducted every 2 years by the National Institute for Health Development (NIHD) in Estonia, and this is a postal questionnaire with a stratified random sample of 5000 people aged 16−64 years (Tekkel & Veideman, 2015). We used responses from the 2014 survey as the control sample for our stroke cohort; individuals aged <18 years or ≥55 years or with a history of stroke were excluded. This study was approved by the Research Ethics Committee of the University of Tartu (302/M‐23).

To evaluate composite health behavior and develop the Health Behavior Stroke Risk Score (HBSR score), we analyzed five factors that have been correlated with an increased stroke risk: smoking status, body mass index (BMI), physical activity, diet, and alcohol use. High‐risk health behaviors were defined based on the recommendations of the NIHD Estonia (Tekkel & Veideman, 2015), and we included seven variables: daily smoking, BMI ≥30, exercise < twice/week, adding salt to ready‐made meals, vegetable consumption <6 days/week, alcohol use frequency once a week or more, and alcohol amount in 7 days >8 units/female or >16 units/male. To calculate the HBSR score, the health behavior factors were graded according to the risk level: low, moderate, or high. Next, the total score (0–10) for each patient was calculated, with a score of 10 corresponding to the highest risk. The description of the score is shown in Table S1. For a binary variable, scores were divided into low (0−5) and high (6−10).

### 3.1 Statistical methods

Comparisons between patients with stroke and controls were performed using the Z‐test, and Bonferroni correction was used for multiple comparisons. The odds ratios (ORs) for each behavioral factor were calculated using logistic regression, presented in both crude and adjusted models, expressed with 95% confidence intervals (CI). The OR for having a high HBSR score was calculated using the same method, and the mean scores were compared using a two‐tailed *t*‐test. All statistical analyses were performed using Stata version 16.1 (StataCorp, College Station, TX, USA).

## 4 RESULTS

**FIGURE 1:**
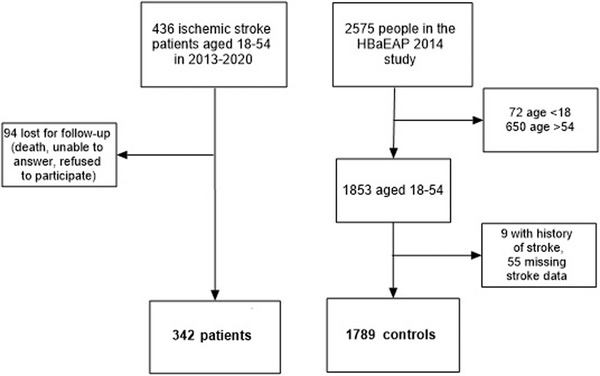
Flowchart depicting recruitment of participants.

In all, 436 young patients with ischemic stroke were recruited in the registry between January 1, 2013 and December 31, 2020. Of these, 342 (78.4%) completed the health behavior questionnaire. There were no statistically significant differences between the respondents and non‐respondents with respect to the distribution of sex. However, the respondents had milder strokes (mean National Institutes of Health Stroke Scale [NIHSS] 4.3 vs 8.8, *p* < .001) with 67.3% having mild strokes (NIHSS ≤ 4), 29.5% moderate (NIHSS 5−15), 2.9% moderate to severe and 0.3% severe strokes. They were also younger (mean age 44.5 vs. 45.7, *p* < .001). A total of 342 patients and 1789 general population controls aged 18−54 years were included in the analysis (Figure 1). Among the patients, there was a higher proportion of males (62% vs. 41%, *p* < .001) and a higher median age (47 years [IQR 39−51] vs. 37 years [IQR 28−46]) than among controls. There were significantly more participants with higher education among controls (34% vs. 20%; *p* < .001), and many of them were single (22% vs. 15%; *p* = .005).

### 4.1 Health behavior

Table 1 shows the prevalence of behavioral risk factors among patients and controls by two age groups. Overall, the proportion of daily smokers was 49.0% among patients and 22.7% in controls (*p* < .001). In addition, the patients were more often obese (33.4% vs. 15.9%, *p* < .001), physically inactive (72.2% vs. 60.5%, *p* = .000), used excessive salt (8.6% vs. 4.5%, *p* = .002), and consumed alcohol at least once a week (39.9% vs. 34.0%, *p* = .009). The differences in vegetable and alcohol consumption over the last 7 days were not statistically significant.

**TABLE 1:** Health behavior of young patients with stroke and the general population controls by two age groups

	Age 18−44			Age 45−54		
	Patients (*n* = 135)	Controls (*n* = 1263)	*p*‐Value^1^	Patients (*n* = 206)	Controls (*n* = 526)	*p*‐Value^1^
Smoking status, *n* (%)						
Daily smoker	62 (46.6)	254 (20.3)	.000*	103 (50.3)	148 (28.5)	.000*
Occasional smoker	10 (7.5)	112 (9.0)	.576	16 (7.8)	33 (6.3)	.481
Ex‐smoker^2^	17 (12.8)	344 (27.5)	.000*	48 (23.4)	133 (25.6)	.545
Non‐smoker	44 (33.1)	539 (43.2)	.025	38 (18.5)	206 (39.6)	.000*
BMI, *n* (%)						
BMI <25.0	53 (40.8)	740 (59.2)	.000*	45 (23.1)	197 (37.8)	.000*
BMI 25−29.9	45 (34.6)	352 (28.1)	.120	74 (37.9)	202 (38.8)	.840
BMI ≥30.0	32 (24.6)	159 (12.7)	.000*	76 (39.0)	122 (23.4)	.000*
Physical exercise, *n* (%)						
≥4 Times a week	16 (12.6)	189 (15.1)	.445	15 (7.9)	66 (12.7)	.077
2−3 Times a week	24 (18.9)	344 (27.5)	.036	32 (16.9)	99 (19.1)	.516
Once a week	18 (14.2)	152 (12.2)	.513	23 (12.2)	68 (13.1)	.743
2−3 Times a month	17 (13.4)	158 (12.7)	.813	16 (8.5)	48 (9.2)	.748
A few times a year or not at all	52 (40.9)	406 (32.5)	.055	103 (54.5)	238 (45.9)	.042
Vegetable consumption, *n* (%)						
≥6 Days a week	51 (39.5)	537 (42.7)	.490	87 (43.7)	226 (43.5)	.950
<6 Days a week	78 (60.5)	721 (57.3)		112 (56.3)	294 (56.5)	
Salting meals, *n* (%)						
Yes, mostly before tasting the food	9 (6.9)	66 (5.2)	.433	19 (9.9)	15 (2.9)	.000*
Yes, when the food needs more salt	86 (65.6)	782 (62.1)	.426	118 (61.5)	346 (66.0)	.256
No, never	36 (27.5)	411 (32.7)	.229	55 (28.6)	163 (31.1)	.526
Alcohol use, n (%)						
≥2 Times a week	26 (19.9)	232 (18.5)	.713	54 (26.9)	92 (17.6)	.006*
Once a week	20 (15.3)	206 (16.4)	.727	33 (16.4)	73 (14.0)	.407
2−3 Times a month	34 (25.9)	334 (26.7)	.859	38 (18.9)	145 (27.8)	.014
Only a few times a year	38 (29.0)	379 (30.3)	.764	51 (25.4)	170 (32.6)	.060
None	13 (9.9)	101 (8.1)	.462	25 (12.4)	42 (8.0)	.068
Alcohol use (last 7 days), *n* (%)						
Excessive alcohol use^3^	23 (18.2)	174 (13.9)	.187	20 (10.5)	64 (12.4)	.491
No excessive alcohol use	103 (81.8)	1075(86.1)		171 (89.5)	454 (87.6)	

Abbreviation: BMI, body mass index.

^a^ Bonferroni corrections were applied to determine the statistical significance of Z‐test *p*‐values, significance marked with an asterisk.

^b^ Ex‐smoker: had quit smoking at least 1 year ago.

^c^ Excessive alcohol use: ≥8 standard drink units/females, ≥16 standard drink units/males.

In further analysis, the odds of having high‐risk health behavior were calculated, and crude and adjusted (age, sex, education, marital status) ORs are presented in Table S2. After adjusting for age and sex, patients with stroke had higher odds (OR [CI]) for daily smoking (2.54 [1.97−3.28]), obesity (1.94 [1.47−2.56]), lack of regular exercise (1.36 [1.03−1.79]), and an unhealthy diet (2.42 [1.34−4.38]), but not for excessive alcohol use. After further adjustments for education level and marital status, the only factors that remained significant were daily smoking (2.13 [1.62−2.80]) and obesity (1.81 [1.35−2.41]). However, a trend did exist with the previously significant factors. When applying the fully adjusted models to the two age groups (18−44 and 45−54) separately, the differences between the age groups were not statistically significant.

### 4.2 Health behavior stroke risk score

**FIGURE 2:**
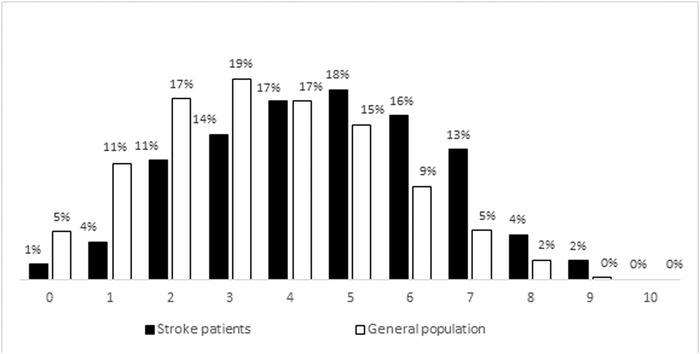
Distribution of the Health Behavior Stroke Risk Score in young patients with stroke and the general population of Estonia.

In young patients with stroke, the average HBSR score was significantly higher than that in the general population (4.6 points [CI 4.4−4.8, SD ± 1.97] vs. 3.5 points [CI 3.4−3.6, SD ± 1.90]; *p* < .001). The distribution of HBSR scores for patients and controls is shown in Figure 2. The prevalence of a score of 6−10 was significantly higher among the patients (52.5% vs. 30.4%, *p* < .001), and this difference was significant for both males (64.3% vs. 45.2%, *p* < .001) and females (33.9% vs. 20.3%, *p* = .001), as well as age 18−44 (42.2% vs. 27.0%, *p* = .001) and age 45−54 (59.5% vs. 38.8%, *p* < .001). The total HBSR score in patients with stroke remained significantly higher after adjustment for age, sex, education, and marital status (Table S2).

## 5 DISCUSSION

Our prospective study showed that young patients displayed significantly worse pre‐stroke health behavior than the general population in most of the studied domains. Following adjustments for confounding factors (age, sex, education, and marital status), smoking and obesity were more prevalent among patients with stroke than controls. The HBSR score provides comprehensive behavioral stroke risk assessment through an aggregate score that was higher in patients than in controls, indicating worse health behavior.

There are minimal reports that have focused on the health behavior of young patients with ischemic stroke, and there is a lack of case–control studies. Our results are in line with those of previous studies reporting smoking and physical inactivity as the most common behavioral risk factors among young patients with stroke (Putaala, 2016). However, while the earlier studies frequently assessed smoking status (Aigner et al., 2017; Goeggel Simonetti et al., 2015; Kivioja et al., 2018; Mitchell et al., 2015; Putaala et al., 2012; Renna et al., 2014; Von Sarnowski et al., 2013), physical inactivity was rarely reported. The most common risk factor in our study was physical inactivity, with more than 72% of the patients and 61% of controls engaging in exercise less than twice a week. Though this criterion is even less strict than that presented in stroke primary prevention guidelines (Meschia et al., 2014), it only involves physical activity during exercising and does not account for action at work or during the commute. In Germany, 49% of young patients with stroke had low levels of physical activity (20−30 min <3 times a week), compared to 19% of controls (Aigner et al., 2017). In addition, a recent study found that excess sedentary leisure time (≥ 8 hours/day) was associated with an increased risk of long‐term stroke in young adults (Joundi et al., 2021).

In this study, almost half of the patients were daily smokers compared with less than a quarter of controls. The difference between our patients and controls was higher than that in similar studies from Germany (48% vs. 35%) (Aigner et al., 2017), Finland (44% vs. 31%) (Kivioja et al., 2018), and the United States (45% vs. 29%) (Mitchell et al., 2015). In the general population, the prevalence of smoking has declined by almost half during the last two decades in Estonia, as it was 18% in 2020 (Reile & Veideman, 2021); however, it remains high in young patients with stroke (Vibo et al., 2021). Based on the high prevalence of smoking among patients, regular documentation of smoking status and cessation support is vital for both primary and secondary prevention.

The weekly alcohol consumption was observed in 40% of the patients and 34% of controls; the difference was statistically significant only before adjusting for age and sex. While differences in the frequency of alcohol intake were noted, the average amount of alcohol consumed (in the last 7 days) was similar in the two groups. The definition of excessive alcohol consumption differs between studies. When defined as >5 alcoholic drinks per day or occasion at least once a month, heavy episodic consumption was recorded in 33% of patients versus 18% of controls in a German sub‐cohort of the SIFAP1 study, and the overall prevalence was very similar (33%) in the multinational SIFAP1 study (Aigner et al., 2017). Heavy drinking, defined as an estimated intake of >200 g of pure alcohol per week, has been reported in 14% of patients with stroke in Finland (Putaala et al., 2009). Similarly, in our previous study, 16% of patients reported the constant use of alcohol (Vibo et al., 2021).

The obesity rate of our patients (33%) was twice as high as that of controls, and it was higher than in most earlier studies of young patients with stroke (11%−22%) (Aigner et al., 2017; Putaala et al., 2012; Renna et al., 2014; Von Sarnowski et al., 2013). To date, only one study has shown a higher prevalence (39.5%), but there was also notably more obesity among controls (29%) (Mitchell et al., 2015). The rate of obesity may reflect the proportion of obese population in a specific country, and the prevalence of obesity is increasing in Estonia (Reile et al., 2020). While BMI is not a behavioral factor in itself but only reflects excessive caloric consumption, we decided to use this factor in our study because it seems to be critical considering the increase in obesity and there is lack of standards for measuring excessive caloric consumption itself.

It is challenging to evaluate diet using only a few questions. We analyzed frequent vegetable and low salt consumption as indicators of a healthy diet. The lack of differences in vegetable consumption between patients and controls was unexpected, as consuming more vegetables has been associated with lower stroke risk in long‐term prospective studies (Aune et al., 2017). It is possible that recall bias may lead to errors in reporting vegetable consumption frequency and that social desirability bias causes patients to report their behavior toward healthier at the hospital (compared to anonymous replies mailed by controls). As for salt use, we found no studies that focused on the habit of adding salt to ready‐made meals or any studies on salt consumption, specifically in young patients with stroke. As per an estimate, patients who add salt to food before tasting consume approximately 10 g of salt per day, while the body requires ∼0.5 g (Spence, 2019). The stroke primary prevention guidelines recommend a diet low in sodium and rich in fruits and vegetables (Meschia et al., 2014). Considering that a healthy diet could notably lower stroke risk (Spence, 2019), clear dietary advice for patients with stroke is needed. It is possible that the effects of diet could not be found in our study due to the young age of the patients, as the health effects of diet accumulate over a long time.

Behavioral risk factors in young patients with stroke have been understudied. Having simple indicator questions for complex variables such as physical activity or diet would facilitate relevant data collection in future research and clinical practice. To summarize the risk factors, we created the HBSR score that is quick and easy to administer, focusing on five important health behavior aspects of stroke in young adults. The questionnaire has only nine questions for the person to answer and is an attempt to create a tool that provides an aggregate score of health behavior that can be used for both research and clinical practice. The HBSR score could be the basis for further counselling for health behavior change as it gives the patient a clear estimate of the modifiable risk factor burden.

A population‐based observational study of behavioral risk factors was performed in Canada, and the Stroke Population Risk Tool was developed (Manuel et al., 2015). That score included the same health behavior aspects as our study, but the variables differed. Health behavior and stroke history were documented during a national survey, and individuals were followed for 5‐year stroke incidence, while we used patients with stroke from the hospital who reported their recent health behavior at the time of stroke. Additionally, they did not focus on young patients with stroke, including 20‐ to 83‐year olds (Manuel et al., 2015).

One of the strengths of our study is recording health behavior during the initial days after stroke onset; therefore, the results reflect the behavior immediately before stroke. Our sample is representative, including all consecutive patients from 8 years from a tertiary stroke center and using a population‐based control group. The aggregate score allows comparisons of overall health behaviors and assesses patients’ lifestyles.

A limitation of our study is that we did not use matched controls; however, we used adjusted models to correct for confounding factors. As patients in our sample had milder strokes than non‐respondents, our results might not represent patients with more severe strokes, but this is more likely to decrease the differences between patients and controls than increase them. Social desirability could have affected the answers, as the control subjects answered the questionnaire anonymously, but the patients with stroke did so during their hospital stay. In choosing our indicator variables, we used questions that are easy to answer and identical to the Estonian HBEAP study; however, these are also more robust and not specifically tested in stroke prevention.

In conclusion, our study demonstrated that young patients with stroke displayed significantly worse health behaviors than the general population in Estonia. As young patients have many decades to benefit from behavior change, more emphasis should be placed on informing patients of these modifiable risk factors and helping enforce these changes (e.g., medication and counselling for smoking cessation, guidelines for exercise and diet). The HBSR score can be used to provide feedback to individuals about the degree of modifiable stroke risk factors in their lifestyle or to compare groups. Further studies are required to evaluate the relationship between HBSR score and stroke risk.

## CONFLICT OF INTEREST STATEMENT

The authors declare that there are no conflicts of interest.

### PEER REVIEW

The peer review history for this article is available at https://publons.com/publon/10.1002/brb3.2908

## Supporting information

Online Supplementary Table 1. The categorization of answers to health behavior questionsOnline Supplementary Table 2. Odds ratios (OR) for high‐risk health behavior in patients with stroke compared to the general population controls

## Data Availability

The data are available upon request from the corresponding author (Minni Saapar).
