# Supplementary material for: Health behavior of young patients with ischemic stroke in Estonia: A score of five factors
Source: Brain Behav. 2023 Feb 14;13(3):e2908. doi: 10.1002/brb3.2908 (PMC10013939; doi:10.1002/brb3.2908)
Supplement: Supplementary file 1 — Online Supplementary Table 1. The categorization of answers to health behavior questionsOnline Supplementary Table 2. Odds ratios (OR) for high‐risk health behavior in patients with stroke compared to the general population controls [file BRB3-13-e2908-s001.docx]

**Online supplement for “Health behavior of young stroke patients in Estonia: a score of five factors”**

**Online Supplementary Table 1.** The categorization of answers to health behavior questions

| **Aspect**  question(s) | **Low risk health behavior – 0 points** | **Medium risk health  behavior – 1 point** | **High risk health  behavior – 2 points** |
| --- | --- | --- | --- |
| **Smoking**  Have you ever smoked in your life? | 1. no | 3. yes, currently occasionally  4. yes, used to smoke before | 2. yes, currently every day |
| **Body Mass Index**  Calculated Body Mass Index^1^ | <25 | 25,00–29,99 | ≥30 |
| **Physical activity**  How often in your free time do you exercise for at least half an hour so that you will breathe a bit heavier and sweat a little? | 1. every day  2. 4–6 times a week  3. 2–3 times a week | 4. once a week  5. 2–3 times a month | 6. a few times a year or not at all  7. can’t exercise due to injury or illness |
| **Diet**  In the past seven days, how often did you consume...   ... fresh vegetables  ... cooked vegetables (exc. potatoes)? (1p)  Do you add salt to your meals on the table? (1p) | Fresh vegetables on  6–7 days OR  Cooked vegetables in 6–7 days OR  Fresh vegetables on  3–5 days AND cooked vegetables on 3–5 days  AND  3 no, never | Fresh vegetables on  6–7 days OR  Cooked vegetables in 6–7 days OR  Fresh vegetables on  3–5 days AND cooked vegetables on 3–5 days  OR  3 no, never | Fresh vegetables on less than 6–7 days OR  Cooked vegetables on less than 6–7 days OR  Fresh vegetables on less than 3–5 days AND cooked vegetables on less than  3–5 days  AND  1. yes, mostly before tasting the food  2. yes, when the food needs more salt |
| **Alcohol use**  In the past 12 months, how often did you consume alcoholic drinks? (1p)  Calculated approximate alcohol units for the past  7 days^2^ (1p) | 4. 2–3 times a month  5. a few times only  6. never  AND  Alcohol units ≤ 16  for males / ≤ 8  for females | 1. (almost) every day  2. 2–3 times a week  3. once a week  OR  Alcohol units > 16  for males/ > 8  for females | 1. (almost) every day  2. 2–3 times a week 3. once a week  AND  Alcohol units > 16  for males/ > 8  for females |

^1^ Calculated from the answer to questions „How tall are you (without shoes)?“ and „How much do you weigh (without clothes)?“ ^2^ Calculated from „In the past 7 days, how many glasses or bottles of the following alcoholic drinks did you consume?“ Cider or light alcohol cocktails with tonic (long drinks): 1 bottle (0.3 l) = 1 units; beer: 1 can (0.5 l) = 2 units; wine or sparkling wine: 1 glass (120 ml) = 1.5 units; hard liquor: 1 shot (40 cl) = 1 unit.

**Online Supplementary Table 2.** Odds ratios (OR) for high-risk health behavior in stroke patients compared to the general population controls

|  | **Crude OR  (95% CI)** | **1^st^ adjusted OR  (95% CI)^1^** | **2^nd^ adjusted OR  (95% CI)^2^** |
| --- | --- | --- | --- |
| Daily smoking | 3.26 (2.57–4.15) | 2.54 (1.97–3.28) | 2.13 (1.62–2.80) |
| BMI ≥ 30 | 2.67 (2.05–3.47) | 1.94 (1.47–2.56) | 1.81 (1.35–2.41) |
| Regular exercise < 2 times/week | 1.70 (1.30–2.21) | 1.36 (1.03–1.79) | 1.29 (0.97–1.71) |
| Diet (total) | 2.26 (1.31–3.89) | 2.42 (1.34–4.38) | 1.78 (0.93–3.41) |
| Vegetable consumption < 6 days/week | 1.03 (0.81–1.30) | 0.95 (0.74–1.23) | 0.93 (0.72–1.21) |
| Adding salt without tasting | 1.99 (1.27–3.11) | 1.99 (1.23–3.22) | 1.59 (0.94–2.68) |
| Alcohol (total) | 0.96 (0.66–1.40) | 0.75 (0.50–1.12) | 0.71 (0.47–1.09) |
| Alcohol consumption ≥ 1 time/week | 1.29 (1.02–1.64) | 0.95 (0.73–1.24) | 0.99 (0.76–1.31) |
| Alcohol units in 7 days >16 males/ >8 females | 1.03 (0.73–1.46) | 0.88 (0.61–1.28) | 0.81 (0.55–1.19) |
| Total Health Behavior Stroke Risk Score 6–10 | 2.83 (2.14–3.74) | 1.85 (1.36–2.51) | 1.66 (1.21–2.27) |

^1^ – adjusted for sex and age; ^2^ – adjusted for sex, age, education, and marital status
